# End-of-life management of multiple myeloma patients in the era of CD38 and immunotherapy

**DOI:** 10.3389/fonc.2024.1436587

**Published:** 2024-10-31

**Authors:** Pierre Sesques, Lionel Karlin, Emmanuel Massy, Alizée Maarek, Guillaume Aussedat, Anne Lazareth, Camille Golfier, Fadhela Bouafia-Sauvy, Helene Lequeu, Dana Ghergus, Violaine Safar, Emmanuelle Ferrant, Emmanuel Bachy, Hervé Ghesquières, Cyrille B. Confavreux, Delphine Demangel, Emeline Perrial, Charles Dumontet

**Affiliations:** ^1^ Department of Hematology, Lyon Sud Hospital, Pierre-Bénite, France; ^2^ Department of Rheumatology, Lyon University Hospital, Pierre-Bénite, France; ^3^ Hospices Civils de Lyon, Lyon, France; ^4^ UMR INSERM 1052/CNRS 5286/University of Lyon, Lyon, France

**Keywords:** myeloma, CD38 targeted therapies, end of life, management, curative and prophylactic therapies

## Abstract

**Background:**

In spite of spectacular advances in the treatment of multiple myeloma, a majority of patients will die from this disease or related complications. While a great amount of focus has been dedicated to the development of novel therapies, little attention has been paid to latter stages of patient follow-up.

**Patients and methods:**

In order to describe patient management during this critical period as well as the immediate causes and circumstances of death, we have analyzed a single center series of 100 patients diagnosed with myeloma who died between 2016 and 2021.

**Results:**

Patients received a median of 3 lines of treatment, including 2 during their last year of life. Sixty per cent of patients had received daratumumab. Fifty patients had obtained complete remission or very good partial response at some time during the course of disease but 75 were refractory to the last treatment line. Eighteen patients died while their disease was stable or in remission while 77 had confirmed progressive disease at time of death. Thirty six patients had uncontrolled sepsis, 49 were in renal failure and 24 had hypercalcemia at the time of death. Seventy three patients presented with lymphopenia. Disease progression was documented in a majority of MM patients at the time of death and was associated with disease-related complications in a significant number of patients.

**Conclusion:**

Disease progression remains the main cause of death in patients with multiple myeloma.

## Introduction

Attention in the field of multiple myeloma (MM) therapy has generally focused on novel therapies. The past two decades has been particularly fruitful with the discovery of immunomodulatory agents, proteasome inhibitors, as well as several immunotherapeutic agents such as anti-CD38 antibodies, an anti-BCMA (B Cell Maturation Antigen) immunoconjugate, anti-BCMAxCD3 or GPRC5D (G coupled Protein Receptor 5 Member D)xCD3 bispecifics and anti-BCMA-targeting chimeric antigen receptor T cells (CART cells) (1). It is well known however that patients included in these clinical trials are highly selected and do not represent the majority of patients with multiple myeloma. This is particularly true in elderly patients, who represent the majority of patients afflicted with this disease. In a hospital-based approach, we reported that the median age at diagnosis was 74 years (2). A growing body of literature is analyzing “real life” data, i.e. the actual management of unselected patients who do not participate in clinical trials. A literature search using the terms “real life “ and “multiple myeloma” had 53 hits, mostly focusing on the use of specific therapies or standards of care outside of clinical trials (3, 4).

As life expectancy of myeloma patients has lengthened and therapeutic options have developed, the management of myeloma is evolving with the possibility to offer a growing number of alternatives in the relapse setting. Currently however the majority of patients with a diagnosis of relapsing myeloma are expected to die of their disease or related complications. In order to better describe the management course of these patients and events occurring during the final year of life we have performed a single center retrospective study, focusing on patients who died with a diagnosis of myeloma between 2016 and 2021.

## Patients and methods

To identify relevant patient case files, we first performed an extraction from the hospital database of all patients with a diagnosis of myeloma followed in the Hematology Department who had died between May 2016 and April 2021. Among the 200+ patients identified we then randomly chose 100 patients to perform this study. Patients included in this study had received a diagnosis of multiple myeloma between June 2000 and November 2020. This study was approved by the Ethics Committee of the Hospices Civils de Lyon. Response to treatment was evaluated using IMWG criteria (5).

## Results

The main patients’ characteristics are described in **Table 1**. The number of patients indicated are to be compared to the entire cohort of 100 patients. Unless specified the percentage values are therefore identical to the number of patients indicated. The sex ratio was 1.17. The median age at diagnosis was 69 years (range: 39-88 years) with 34 patients aged 65 or less and 26 aged 75 or more and a median follow-up of 5.3 years. The median age at time of death was 74 years (range: 39-90 years). The most frequent indication invoked for treatment initiation was the presence of bone lesions (n=77), while several patients presented with several CRAB criteria simultaneously (n=51), with anemia being present in 45 patients at diagnosis. Serum albumin levels were reduced (<40 g/L) in 76% of patients for whom this information was available. Data were unavailable to determine IMW risk.

**Table 1:** Patient characteristics at diagnosis.

	N=100
Age at time of diagnosis (yrs) at time of death (yrs)	69 (38-88) 74 (39-90)
Sex ratio (male/female)	54/46
Presence of CRAB criteria for therapy initiation C*alcium* R*enal* A*nemia* B*one*	27 28 45 77
Immunoglobulin subtype A G D M Light chain only Non secretory Light chain subtype Kappa Lambda Unavailable Non secretory	25 42 3 1 26 3 50 45 2 3

The main events occurring during the course of disease are summarized in **Table 2**. Ninety-seven patients of this series had received treatment for myeloma. The three others either did not present with CRAB criteria or had a co-existing disease which took precedence over treatment of myeloma. As an example, one of these three patients presented with inoperable non-small cell lung cancer, received carboplatin/taxol chemotherapy after which he presented a 48% decrease in his serum immunoglobulin component, but died shortly thereafter of his lung disease. Among patients receiving therapy for MM, the time to initiation of first line therapy was relatively short, with a median delay of 31 days after diagnosis (range: 1-7299 days). A majority of the patients received immunomodulatory drugs (IMIDs) (n=60) and/or proteasome inhibitors (n=61) in their first line regimens, while 29 patients proceeded to high dose melphalan therapy with autologous stem cell transplantation (age cutoff of 65 years).

**Table 2:** Therapies and events.

	N=100
Delay between diagnosis and first line (days)	31 (10-1541)
Number of lines of therapy	3 (0-8)
Therapeutic agents received As first line None Steroids Melphalan High dose melphalan with ASCT IMID Proteasome inhibitor Anti CD38 antibody During the course of disease None Steroids Melphalan Proteasome inhibitors IMIDs High dose Mel with ASCT Anti CD38 antibody Other Clinical trial Radiotherapy	3 97 23 29 60 61 1 3 97 55 87 88 29 58 30 35 23
Best response to therapy CR VGPR PR SD PD Non evaluable	16 34 32 6 2 10
Complications during course of disease C (hypercalcemia) R (renal failure)/dialysis A (anemia) B (bone disease) Amyloidosis DVT/PE PCL	37 45/8 77 78 (11 plasmocytomas) 4 5 4

CR, complete response; DVT, deep vein thrombosis; PCL, plasma cell leukemia; PE, pulmonary embolism; PR, partial response; SD, stable disease; VGPR, very good partial response; IMID, immunomodulatory drugs; Mel, Melphalan.

During the course of their disease, a large majority of the patients received an IMID (88 patients) and/or a proteasome inhibitor (87 patients) and 58 patients received an anti-CD38 antibody at some time during their management. All patients received high dose steroids. Thirty patients received other therapies, including doxorubicin, vincristine, bendamustin or experimental compounds. Thirty-five patients participated in clinical trial at least once during the course of their disease. Twenty-three received radiotherapy and 8 required dialysis. Among the 90 patients who were evaluable for response, 50 (56%) obtained complete response or very good partial response at some point during their management. Only 2 progressed on all therapies administered while the rest had a partial response or stable disease.

During the course of their disease, 78 patients presented recurrent or developed symptomatic bone lesions, while 37 had hypercalcemia. Forty-five suffered from renal failure, including 8 patients who underwent dialysis. Seventy-seven patients had symptomatic anemia (hemoglobin< 90 g/L). Eleven patients developed plasmacytomas in various skeletal and non-skeletal localizations. Four patients developed symptomatic amyloidosis. Four patients evolved towards plasmablastic leukemia.

The median time between diagnosis and death in this series was 3.71 years (range 0.07 to 20.7 years) and between first treatment and death was 3.3 years (range 0.01 to 19.5 years). Patients who were treated for their myeloma received a median number of 3 lines of therapy (range 1-8) over a period of 3.26 years (range 0.07 to 17.1 years). The time between the last “curative” therapy (defined as other than exclusive steroids or low dose oral cyclophosphamide) and death was 38 days, with a large range spanning from 1 to 2443 days depending on the quality of response to this line of treatment. In a large majority of patients (n=75) disease was refractory to their last regimen. Overall the “curative therapeutic period”, i.e. the period extending from the first line of therapy to the last curative treatment lasted for a median period of 3.15 years (range 0.01-17.1 years).

Follow-up during the last year of life is detailed in **Table 3**. A majority of patients received prophylactic and/or symptomatic therapies during their last year of life, per institutional protocol. Sixty-eight patients received prophylactic anticoagulants or antiagregants and 74 prophylactic antibacterials or antivirals. Fifty-two received red blood cell transfusions, thirty-one platelet transfusions and fourty-four hematopoietic growth factors. Forty-nine patients required curative anti-infectious agents, a majority of which were administered in the in-patient setting. During this period 66 received stage 2 or 3 painkillers.

**Table 3:** Patient follow up during the last year of life.

	N=100
**Localization at death** Home Local hospital Geriatrics Palliative care Hematology/specialized department ICU	20 8 12 20 25 15
**Time between last treatment and death (days)**	38 (1-2443)
**Number of imagery exams during last year**	7 (0-25)
**Treatment received during last year** Steroids and palliative Anticoagulants/antiagregants Antiinfectious (preventive) Biphosphonates Growth factors RBC transfusions Platelet transfusions IMIDs Proteasome inhibitors Melphalan High dose Mel with ASCT Anti CD38 Clinical trial Anti-infectious (curative) Painkillers	92 68 74 31 44 52 31 71 54 2 1 44 5 49 66
**Response to last therapy** CR/VGPR PR SD PD Non evaluable	5 7 8 75 5
**Disease status at death** Remission/stable Progressive Unknown	18 77 5
**Biology at time of death** Hypercalcemia > 2.5 mM Anemia (Hb<90g/L) Renal failure (creat >110) Neutropenia Lymphopenia < 1 giga/L Thrombocytopenia < 50 giga/L	24 52 49 17 73 40
**Immediate cause of death£** Myeloma progression Other disease, including: Other neoplasia Neurological diseases Infection DVT Treatment related toxicity	78 23 7 4 36 (including 4 Covid) 3 32

CR, complete response; DVT, deep vein thrombosis; PR, partial response; SD, stable disease; VGPR, very good partial response; ICU, intensive care unit; RBC, red blood cell; IMID, immunomodulatory drugs; Mel, Melphalan. £: the total value of causes exceeds 100 as several causes were deemed involved in occurrence of death in certain patients.

In the days preceding their death a majority of evaluable patients (78/95, 82%) had evidence of progressive disease. Twenty-four presented with hypercalcemia, 52 had symptomatic anemia and 49 had renal failure. Seventeen had profound neutropenia, 40 had thrombocytopenia and a majority (73%) were lymphopenic. Identifiable causes of death included progressive myeloma (n=78), uncontrolled infection (36, including 4 with COVID) and deep venous thrombosis or pulmonary embolism (n=3). Thirty-two were considered to suffer from treatment related toxicity, consisting mainly in some form of myeloid toxicity, contributing to treatment discontinuation and infectious complications. One patient with stable myeloma died of secondary AML.

## Discussion

Management of patients with myeloma requires curative as well as symptomatic and prophylactic therapies adapted to the various stages of the disease. We therefore performed a retrospective analysis of patients followed for multiple myeloma in our tertiary center and who had died over the past five years in the era of CD-38 targeted therapies. Importantly our retrospective study encompasses a period of observation during which CD38-targeted therapies were only available in the relapse setting for some of the patients. We examined the nature of the treatments administered, response achieved, events occurring during the course of disease and particularly focused on the last year of life.

In this unselected population the median age at diagnosis was 69 years. This value, which is 5 years lower than what we previously reported at the nation-wide level, is common in teaching hospitals such as our center. Patient characteristics were unremarkable, with a higher male/female ratio of 1.17 and a classical Ig subtype distribution. The overall survival observed in this unselected group after the initiation of first therapy is relatively short with a median value of 3.3 years, but quite similar to that reported in the Ontario cohort (3.6 years from diagnosis to death) (6).

Among the 97 patients who received specific therapy for myeloma, two thirds had received an IMID or a proteasome inhibitor as first line. During the course of disease a large majority of patients received this type of agents and 58 also received an anti-CD38 antibody at some time during their management. Of note, 35 patients participated in a clinical trial, either for first line therapy or at relapse, which favored their access to novel therapies or therapeutic strategies. This relatively high participation rate is due to the teaching hospital status of our center. Only twenty-nine patients underwent high dose therapy with autologous stem cell transplantation, which is in keeping with the fact that 33 patients were aged 65 or less at time of first line therapy. As a whole, these patients had a relatively early access to novel therapies, and that the main families of active agents were available during the course of disease. As CD38-targeted therapies are currently available in the first-line setting it will of particular interest to compare the results of our study with those of patients receiving targeted therapies in first line.

Remarkably the last “curative” treatment (defined as a therapy other than steroids and/or low dose oral cyclophosphamide) was administered in average up to 38 days before death. This period was not much longer in patients over or under the age of 65 (42 vs. 34 days). The median number of lines during the last year of life was 2 (range 0-5) in patients who had received therapy for myeloma. A majority of patients followed in our center thus received therapies commonly administered with curative intent shortly before death. This is similar to the observations made in another French center but in contrast to the results reported by McInturf et al. who found that 33.5% of patients had received non-palliative therapy during the month before death (7, 8). In a population-based cohort followed in Ontario between 2006 and 2018, 23.2% of patients were reported to have received chemotherapy at end-of-life (6).

The best response observed during the course of therapy was CR or VGPR in 56% of patients. This value is expected to increase thanks to recently discovered innovative therapies or better use of existing agents. Only two patients were considered to be primary refractory to therapy. Conversely patients developed chemoresistance during the course of their disease, with 79% of patients progressing after their last curative treatment. Of note however 19% were considered to have stable or responding disease at time of death. Alternative causes of death in patients who did not have progressive myeloma were diverse, including 7 solid tumors, 4 neurological diseases and other preexisting comorbidities.

Various complications, in particular infectious and thrombo-embolic complications, commonly occur in myeloma patients during the course of disease, with a contributive role of therapy. Adapted prophylactic therapy is therefore required to avoid complications, dose reductions or treatment discontinuation. The impact of novel therapies on the rate of high-grade infection remains a subject of controversy (9). Prophylactic valaciclovir is recommended in European guidelines in high-risk patients (10). Intravenous immunoglobulins have been proposed as a prophylactic measure in patients who reach a plateau-phase, with a strong protective effect, or in the stem cell setting, with less convincing results (11, 12).Thrombo embolic complications are a major concern in myeloma patients, with both a predisposition to increased risk due to disease and a role of certain therapies such as IMIDs (13, 14). Prophylactic therapy is widely used in patients receiving IMID based therapies, although the optimal type of prophylaxis remains open to debate (15). Anemia is commonly observed in myeloma patients and 52 patients in our series required RBC transfusions, a value similar to that reported in the Kansas series (46.3%) (7).

Regarding localization at time of death, only 20% of patients were at home at time of death. Forty patients were in a palliative or geriatric setting while the other 40 patients were in a specialized department, including 15 in intensive care settings. Many of these patients were rehospitalized during their last days of life as management proved to be too complex at home, and a number of acute events required hospitalization. In the Ontario cohort 55.6% of the patients died as inpatient (6). Interestingly Abbasi et al. compared patients who died in 2002 and 2017 and found that patients were increasingly managed in palliative care or through hospice consultations while a smaller percentage was dying in the hospital (16). Patients who were hospitalized tended to have more transfusion requirements and/or a higher infection burden. A similar increase in hospice use was observed in a large retrospective U.S. cohort based on SEER data (17).

Identifying the immediate causes of death in myeloma patients is complex since these patients are likely to suffer from: a) disease progression, along with hypercalcemia, bone fractures, symptomatic plasmacytomas or plasmablastic leukemias; b) disease-related complications, including renal failure, infections or thrombo-embolic events, c) therapy-related complications; d) comorbidities. Additionally, patients who are in a palliative environment have less blood tests and exams and it is therefore difficult to determine what is at stake during the final days of life. To address this issue, we examined patient files and extracted the biological results performed closest to death. According to the referent physician, myeloma progression was a direct cause of death in 78 patients. Thirty-six had uncontrolled infection at time of death and thirty-two presented with treatment-related toxicity, consisting mainly in myeloid cytopenias. Interestingly a majority (73%) were lymphopenic at time of death, which is likely to be a predisposing factor for severe sepsis. Half of the patients (49%) had renal failure and quarter (24%) had some degree of hypercalcemia. A majority (66%) were receiving potent painkillers at time of death. In the Kansas series disease progression was present at death in 77.4% of patients (7).

A limitation of this study is that it was performed retrospectively in a single center teaching hospital. However, a number of our observations are likely to be transposable to other settings. In particular our study shows that a majority of patients with myeloma currently die in the hospital or palliative setting with progressive disease. Pain management remains a critical issue during this period with 2/3 of patients requiring major pain-killers, and is an important cause for rehospitalization of patients who were at home. In the Kansas series 84.2% of patients had received opioid medication in the year prior to death (7). This requirement for strong antipain medications is largely, but not exclusively, due to the high prevalence of active bone lesions in these patients. Uncontrolled infection was involved in a third of patients in spite of widely used prophylactic therapies. Our center does not routinely administer substitutive immunoglobulin therapy in the palliative setting. Renal failure was a contributing factor in half of the patients and hypercalcemia was observed in ¼ of the patients. Conversely thrombo-embolic events, which may have been underdiagnosed in our series, were relatively infrequent and confirmed in 3 patients in our series. A striking observation was the relatively high occurrence of symptomatic and multiple plasmacytomas in 11 patients, including a number of various localizations such as orbit, peritoneum, pancreas or skin.

These observations emphasize that the main cause of death is lack of disease control, with a frequent contribution of disease-related complications and treatment-related complications. Symptomatic measures aiming to better control pain, severe sepsis, cytopenias and hypercalcemia would contribute to improved quality of life in the final stages of this disease. Plasmacytomas compromise quality of life in a substantial fraction of patients. The frequent administration of non-palliative therapy in the months preceding death suggests that a better evaluation of its benefit, or a more precise identification of the subgroup of patients for whom it is beneficial, would be useful.

## Data Availability

The data analyzed in this study is subject to the following licenses/restrictions: Access to be requested to the Hospices Civils de Lyon. Requests to access these datasets should be directed to charles.dumontet@chu-lyon.fr.
